# Compositional and operational impacts on the thermochemical reduction of CO_2_ to CO by iron oxide/yttria-stabilized zirconia†

**DOI:** 10.1039/d0ra08589h

**Published:** 2021-01-05

**Authors:** Eric N. Coker, Andrea Ambrosini, James E. Miller

**Affiliations:** Sandia National Laboratories P.O. Box 5800 Albuquerque NM 87185-1411 USA encoker@sandia.gov; LightWorks®, Arizona State University Tempe AZ 85281 USA

## Abstract

Ferrites have potential for use as active materials in solar-thermochemical cycles because of their versatile redox chemistry. Such cycles utilize solar-thermal energy for the production of hydrogen from water and carbon monoxide from carbon dioxide. Although ferrites offer the potential for deep levels of reduction (*e.g.*, stoichiometric conversion of magnetite to wüstite) and correspondingly large per-cycle product yields, in practice reactions are limited to surface regions made smaller by rapid sintering and agglomeration. Combining ferrites with zirconia or yttria-stabilized zirconia (YSZ) greatly improves the cyclability of the ferrites and enables a move away from powder to monolithic systems. We have studied the behavior of iron oxides composited with YSZ using thermogravimetric analysis under *operando* conditions. Samples in which the iron was fully dissolved within the YSZ matrix showed greater overall extent of thermochemical redox and higher rate of reaction than samples with equal iron loading but in which the iron was only partially dissolved, with the rest existing as agglomerates of iron oxide within the ceramic matrix. Varying the yttria content of the YSZ revealed a maximum thermochemical capacity (yield per cycle) for 6 mol% Y_2_O_3_ in YSZ. The first thermochemical redox cycle performed for each sample resulted in a net mass loss that was proportional to the iron oxide loading in the material and was stoichiometrically consistent with complete reduction of Fe_2_O_3_ to Fe_3_O_4_ and further partial reduction of the Fe_3_O_4_ to FeO. Mass gains upon reaction with CO_2_ were consistent with re-oxidation of the FeO fraction back to Fe_3_O_4_. The Fe dissolved in the YSZ matrix, however, is capable of cycling stoichiometrically between Fe^3+^ and Fe^2+^. Varying the re-oxidation temperature between 1000 and 1200 °C highlighted the trade-off between re-oxidation rate and equilibrium limitations.

## Abstract 2

The versatile redox chemistry of ferrites makes them useful as active materials for the solar-thermochemical production of synthetic fuels. Optimization of the distribution of iron in a YSZ matrix allows the performance of ferrites to be enhanced.

## Graphical abstract

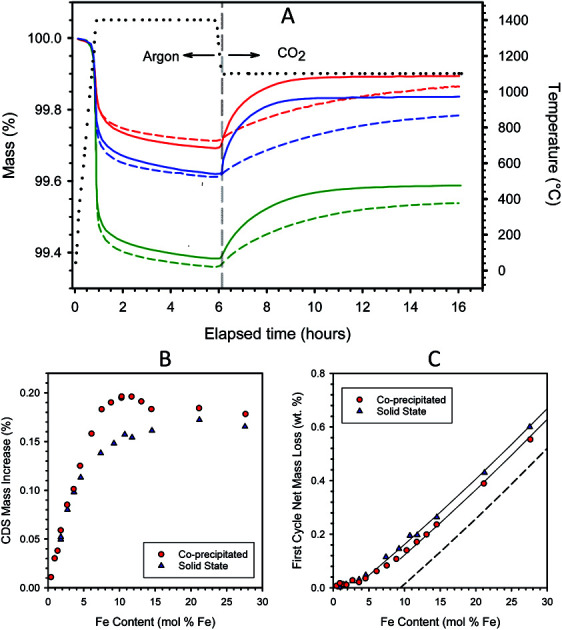

## Introduction

Solar-driven two-step ferrite thermochemical cycles are of interest as a method for producing H_2_ and CO *via* H_2_O- and CO_2_-splitting^1–5^ due to the moderate temperatures required for thermal reduction and large change in solid state oxygen content between reduced and oxidized state, granting them high gravimetric capacity for H_2_ and CO production. Recently non-stoichiometric oxides such as ceria (CeO_2_) have been investigated as redox-active materials for two-step thermochemical cycles because of their fast redox kinetics, high fuel selectivity, and retention of phase stability after thermal reduction.^6–8^ Unfortunately, ceria requires extremely high temperatures around 1550 °C for reduction to a commercially viable extent,^9,10^ which presents many engineering and materials challenges to developing a working thermochemical engine.^11,12^ Attempts to alleviate ceria's thermal reduction limitation have mostly been unsuccessful,^13–18^ and new redox materials that operate effectively at lower thermal reduction temperatures are sought. One class of materials that has received attention is the perovskite oxides,^19^ which have been applied in related fields where oxygen exchange is required, *e.g.*, solid oxide fuel cells,^20^ chemical looping,^21^ and electrochemical water splitting.^22^ Several perovskites including Sr_*x*_La_1−*x*_Mn_y_Al_1−*y*_O_3−*δ*_,^23^ La_0.6_Ca_0.4_Mn_1−*y*_Al_*y*_O_3−*δ*_,^24^ and BaCe_0.25_Mn_0.75_O_3−*δ*_ ^25^ have been reported as potential new thermochemical materials. Despite the enhanced rates of redox shown by non-stoichiometric oxides compared to iron-based systems, ferrites are still of interest due to the potential to achieve deeper levels of reduction; the challenge remains to enhance the rates of redox in such materials.

The most basic Fe_3_O_4_ ferrite cycle includes a thermal reduction step (TR; reaction (1)) in which solar thermal energy reduces Fe^III^ to Fe^II^, and the ferrite spinel structure transforms to wüstite (FeO). This is followed by a water-splitting step (WS; reaction (2)), or carbon dioxide-splitting step (CDS; reaction (3)) wherein the spinel is regenerated:

1 Fe_3_O_4_ → 3FeO + 0.5O_2_

2 3FeO + H_2_O → Fe_3_O_4_ + H_2_

3 3FeO + CO_2_ → Fe_3_O_4_ + CO

However, this Fe_3_O_4_ cycle, originally proposed by Nakamura,^26^ is not practical since, at reasonable oxygen partial pressures, the TR to wüstite requires temperatures in excess of the melting point of both FeO (1377 °C) and Fe_3_O_4_ (1597 °C). Charvin, *et al.*^27^ reported 100% conversion of Fe_2_O_3_ to FeO in 2 minutes at 1700 °C under N_2_ at 0.1 bar. A consequence of the high reduction temperatures is sintering or fusion of the reactive powder that must be reversed by mechanical crushing or milling in order to reactivate the material for the successive reaction with H_2_O or CO_2_, as these reactions are found to be limited to the surface regions of the material at thermodynamically reasonable temperatures. The TR behavior can be manipulated in the ferrite redox system by partial cation substitution of Fe in the spinel,^28^ A_*x*_Fe_3−*x*_O_4_ (A = Mn, Co, Ni, Zn). This can shift the reduction temperatures lower by >100 °C while simultaneously increasing melting points.^29,30^ The reduction temperatures observed in practice are a function of the composition, the oxygen partial pressure over the material, and reaction kinetics, which improve with temperature. Thermal reduction has been reported to occur as low as 700–1100 °C for Ni-substituted and Ni/Mn co-substituted ferrites,^31,32^ though temperatures ≥ 1200 °C, which are achievable using concentrated solar-thermal energy,^29^ are more typically used to drive the reaction. Although the re-oxidation (CDS or WS) reaction becomes more favorable as the temperature decreases (*i.e.*, the equilibrium shifts towards oxidation), reaction kinetics are once again a consideration, and the decrease of oxidation rate with temperature limits the practical lower-end temperature. The ferrite re-oxidation step is thus often carried out within a few hundred degrees of the reduction temperature. This is problematic as the extent of the temperature swing is directly linked to the potential system efficiency.^33^

While substituted ferrites are a significant improvement over the unadulterated system, the gains are only realized in particulates. Monolithic substituted ferrite structures remain largely unreactive.^34^ Kodama, however, pioneered a second approach to improving the ferrite process by demonstrating that supporting Fe_3_O_4_ on zirconia or yttria-stabilized zirconia (YSZ) eliminated the need for mid-cycle mechanical processing,^4,35^ an approach that extends to analogous monolithic systems,^5,36^ which are reactive and stable over extended periods of cycling.

We have shown previously that Fe may be dissolved into 8YSZ (8 mol% Y_2_O_3_ in ZrO_2_) to a level of *ca*. 9.4 mol% Fe and that Fe in this solid solution is more amenable to reduction–oxidation (redox) cycling than Fe that is present as bulk oxide particles embedded in the 8YSZ matrix.^37,38^ Furthermore, it was shown by isotopic labeling that the permeation of oxygen into bulk iron oxide particles was surface-limited, and samples prepared through co-precipitation exhibited better overall permeation of oxygen into the ferrite particles than those prepared by solid state synthesis when the total iron content in the samples was equivalent.^39^ These studies helped to clarify and verify some of the early hypotheses regarding the combined system,^35,40,41^ thereby providing a solid foundation of understanding of the workings of the composite system. In the current work we build upon this foundation but also address the more practical issue of establishing routes for, and the limits of, improvements to the ferrite system *via* compounding with YSZ. In order to study the effect of varying the ratio of dissolved iron to phase-segregated iron independently of total iron content, two synthetic approaches were employed. Furthermore, the effects of composition (iron and yttria concentrations) and operating conditions on thermochemical activity were examined. While prior studies have used *post mortem* analysis of high temperature-treated materials, the data obtained may not accurately reflect the properties of a material under high temperature conditions. As we showed earlier using *operando* characterization methods,^37^ enhanced iron mobility as well as changes in solubility of iron in 8YSZ occur at high temperature as the oxidation state of the iron varies; this data could not be inferred easily from *post mortem* analyses. Thus, we monitor the materials during simulated thermochemical cycling.

## Experimental details

### Materials synthesis

The yttria (Y_2_O_3_) content of YSZ is defined as mol% Y_2_O_3_ in ZrO_2_; thus, 8YSZ has the composition (ZrO_2_)_0.92_(Y_2_O_3_)_0.08_. The Fe content of iron oxide/8YSZ materials is given in mol% of the metals, *i.e.*, 100 × mol(Fe)/(mol(Fe) + mol(Y) + mol(Zr)). Samples were prepared by one of two routes: co-precipitation (CP) or solid state synthesis (SS). All samples were subjected to high-temperature air calcination and sintering to form the thermochemically active material prior to characterization.

#### Co-precipitation (CP)

For CP, stoichiometric molar cation quantities of iron(iii) nitrate (Fe(NO_3_)_3_·9H_2_O, Fisher Certified ACS, 99.8%), yttrium nitrate (Y(NO_3_)_3_·6H_2_O, Alfa Aesar 99.9% (REO)), and zirconyl nitrate (ZrO(NO_3_)_2_, Aldrich, 35 wt% in dilute HNO_3_, 99+%) were combined and dissolved in water at 60 °C. The metal-containing solution was added slowly to a solution of ammonium hydroxide (NH_4_OH, Fisher Certified ACS Plus, 29%, 14.8 N) in water also at 60 °C with stirring. The quantity of NH_4_OH was such that the concentration of OH^−^ exceeded the concentration of NO_3_^−^ as required for complete precipitation of the metals. Quantitative chemical analysis by Atomic Absorption Spectrophotometry of post-precipitation supernatant solutions showed insignificant concentrations of Fe, Zr, or Y remaining in solution, verifying that the extent of precipitation was complete, within experimental error. The precipitate thus formed was filtered, washed, and dried in a forced convection oven at 60–80 °C. Powder samples were mixed with an organic binder consisting of 3.5 wt% polyvinyl alcohol and 3.5 wt% polyethylene glycol dissolved in water. The powders were mixed thoroughly with the solution to make a thick paste which was then dried at 60 °C, ground in a mortar and pestle, and formed into 12 mm diameter circular discs of approximately 1 g in an isostatic pellet press at 3 tons. The binder was burned out in a vented furnace by heating in air from room temperature to 600 °C at 5 °C min^−1^ and holding for 2 hours. The resulting pellet was then sintered in air at 1350 °C for 36 hours and 1450 °C for 4 hours before cooling to room temperature. All ramp rates were set to 5 °C min^−1^ (nominal).

#### Solid state synthesis (SS)

The solid state synthesis procedure has been described in an earlier publication.^37^ Briefly, hematite powder (Fe_2_O_3_, Fisher, 99.5%) was ground with 8YSZ powder (Tosoh Corporation, 99.9%) in a mortar and pestle and mixed thoroughly. The addition of binder, disc formation, binder burn-out, and final sintering were identical to those described for the CP materials.

Preliminary data indicated that fusion and volatility of iron oxides can occur at 1500 °C,^42^ and the intermediate soak at 1350 °C during sintering was to allow iron species to diffuse into the 8YSZ and form a solid solution prior to the final sinter at 1450 °C [Fe_2_O_3_ melting point: 1566 °C (decomp.); Fe_3_O_4_ melting point: 1597 °C; FeO melting point: 1377 °C].

### Characterization

Room temperature XRD patterns were recorded for finely-ground powders on either a PANalytical X-Pert Pro diffractometer or a Siemens D500 diffractometer using Cu Kα radiation.

Thermogravimetric analysis (TGA) was carried out using a Netzsch STA 449 F3 Jupiter, as described in detail elsewhere.^37^ Briefly, thermochemical cycling experiments in the TGA were conducted under heating and cooling rates of 25 °C min^−1^ and gas flow rates of 140 ml min^−1^. Two distinct experimental protocols were used during characterization by TGA, one to determine the maximum CO yield per gram of solid achievable (within a reasonable timeframe) and one to determine the rates of reaction. The dense monolithic sample pellets were analyzed as-prepared, or broken into 4–8 fragments, depending on the mass required. None of the data presented here was measured on a powder material.

To determine CO_2_ to CO conversion maxima, long reaction times and relatively large specimens were used in the form of sintered discs, or fragments of discs, of reactive material (disc dimensions approx. 12 mm diameter, 2–3 mm thick, weighing ∼400–800 mg) placed on top of a platelet of single-crystal 8YSZ (MTI Inc.) to prevent reaction with the Al_2_O_3_ specimen holder. The specimen was heated under Ar to 1400 °C, held isothermally for 5 hours (thermal reduction), cooled to 1200 or 1100 °C, exposed to CO_2_ (120 ml min^−1^ CO_2_ with 20 ml min^−1^ Ar), and held isothermally for 10 hours (re-oxidation). Additionally, some samples were thermally reduced as above and re-oxidized under CO_2_ at 1000 °C for 15 h. Each sample was analyzed in the TGA at least twice, and the sample was re-weighed between runs.

Details of control experiments conducted to ensure that mass transfer limitations did not influence the results discussed here are given in the ESI (Section S1 and S2).†

Specimens for rate determination *via* TGA were fragments of sintered discs of reactive material weighing between 100 and 120 mg. Specimens were heated to 1400 °C, held for 30 min, then cooled to 1100 °C, all under flowing Ar. After equilibrating at 1100 °C for 15 min under Ar, the specimen was exposed to CO_2_ to re-oxidize the ferrite for 30 min before switching back to Ar, heating to 1400 °C and thermally reducing for another 30 min. This redox cycle typically was repeated a total of 5 times.

All TGA data are presented as mass% as a function of time, where 100% represents the total specimen mass at the beginning of the run, *i.e.*, without normalizing to the ferrite content of the specimen. The errors associated with sample preparation and thermal analysis were determined to be less than 0.1% (relative) in both iron content and TGA mass change, thus error bars are smaller than the size of data symbols in the figures.

Potential hazard warning: carbon monoxide (CO) is a poisonous gas. Ensure that the gas stream exiting any reaction chamber where CO_2_ splitting is being carried out is directed to an exhaust hood and/or scrubber to remove the CO safely.

## Results and discussion

### Effect of iron loading and dissolution in 8YSZ

#### Cyclic redox reaction extents

**Fig. 1:**
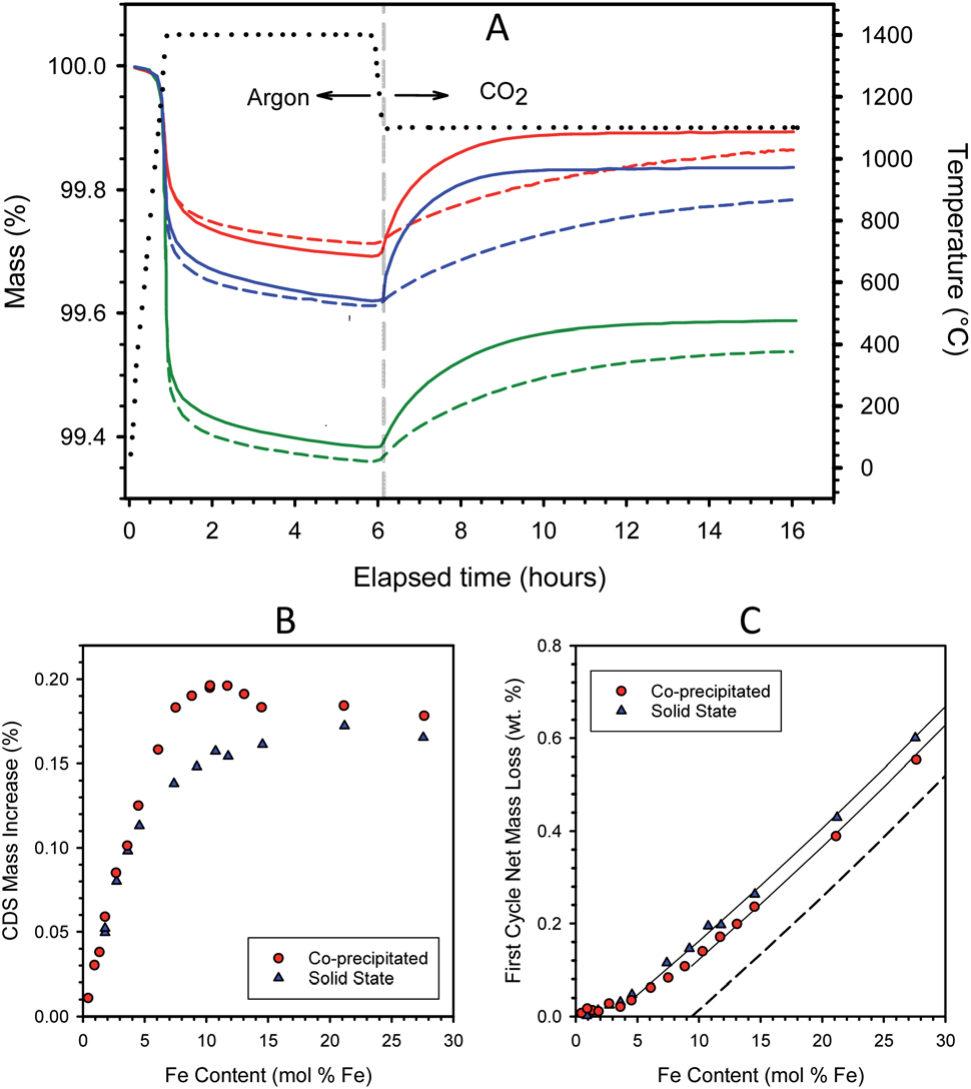
Representative TGA curves measured during a thermochemical redox cycle on freshly calcined materials. Samples prepared by CP (solid lines) and SS (dashed lines) have Fe loadings of 7.5 mol% (red), 10.3 mol% (blue), and 21.2 mol% (green) in 8YSZ. The dotted black curve depicts the sample temperature, and the vertical dashed line indicates where the environment was switched from reducing to oxidizing (A). Mass increase during re-oxidation under CO_2_ as a function of iron loading for samples prepared by the CP and SS routes. Samples were thermochemically cycled as in panel (A) (B). Net mass loss during the 1st complete redox cycle for samples prepared by CP and SS routes as a function of iron loading. Guidelines show the approximately linear relationship between mass loss and iron loading above the point where full solubility is presumed. The dashed line indicates the theoretical mass loss assuming dissolved Fe (below 9.4 mol%) re-oxidizes fully under CO_2_ (C).

Samples containing Fe loadings ranging from 0.5 to 28 mol% Fe in 8YSZ were prepared. The amount of undissolved iron observed *via* XRD and SEM was greater for samples prepared *via* SS than by CP when the total iron content was similar. The results of the thermal redox cycling are strongly dependent on the distribution of iron. For materials with equivalent Fe loadings, the reversible redox capacity increases with the amount of dissolved iron (Fig. 1A). The rate of re-oxidation shows the same trend, *vide infra*. The samples prepared *via* the SS route, which contain a smaller fraction of dissolved iron, only approach a steady-state re-oxidation level after 10 hours, while those prepared by CP reach steady-state within 4 to 8 hours, depending on the Fe loading.

The mass gains observed during re-oxidation are plotted as a function of Fe content in Fig. 1B. The mass increases are measured from the beginning of the CO_2_ exposure period to the end of the 10 h isothermal soak. The data for CP and SS samples fall atop one another below about 4–4.5 mol% Fe. At higher loadings, the CP samples exhibit greater overall redox extents than their SS counterparts. The data trend for the CP samples shows a roughly linear increase in redox capacity with increasing loading up to approximately 8 mol% Fe, above which there is a small plateau to about 10 mol% Fe. Recall that this region corresponds to the composition range (0–9.4 mol% Fe) wherein previous studies confirm most, if not all, of the Fe is atomically substituted into the 8YSZ as a solid solution.^37,38^ The addition of Fe to the CP preparation in excess of 10 mol% not only fails to improve the capacity of the material but decreases it. In contrast, the redox extents of the SS samples increase gradually between 5 and >20 mol% Fe, levelling off at the highest loadings.

The systematic difference in redox performance between the CP and SS is traceable to a combination of chemical and physical differences in the materials, more specifically to dissimilar distributions of Fe throughout the 8YSZ matrix, and between more chemically active/reactive dissolved iron species and less active/reactive secondary iron oxide phase(s). In essence, the CP materials possess a greater proportion of dissolved iron than their SS counterparts of equivalent iron loading. The CP materials begin as a co-precipitated amorphous precursor solid in which inter-mingling of all components is presumably achieved on an atomic scale. In contrast, the SS materials begin as co-mingled but separate and distinct crystalline particles of hematite and 8YSZ. In the CP case, the active dissolved Fe species are expected to form directly upon thermal treatment (dehydration and sintering) and to be homogeneously distributed through the material. Presumably the Fe undergoes principally direct crystallographic substitution onto the YSZ lattice, rather than interstitial incorporation.^43^ Only iron in excess of the solubility limit should precipitate into distinct iron oxide particles, the size and/or initial number of which can be expected to increase with increasing iron concentration. The average size of the excess iron oxide particles will increase over time at high temperature through a process of migration and particle coalescence. The SS samples undergo almost the reverse process upon thermal treatment. The most active dissolved iron species form at high temperature as the result of surface reaction/interaction between iron oxides and YSZ, followed by slow diffusion of atomic iron species through the YSZ crystal lattice. Note that in contrast to the CP synthetic method, wherein bonds are created during the precipitation process, the SS process requires the breaking and remaking of strong metal–oxygen bonds when Fe dissolves into the YSZ matrix. Simultaneously, small iron oxide particles will migrate and coalesce into larger particles, and YSZ grains will sinter and grow in size. Fig. S3 in ESI Section S3† shows representative SEM images and EDS maps for iron distribution in dense monolithic samples containing 4.5 and 14.5 mol% iron, prepared by both CP and SS routes. The two 4.5 mol% Fe samples show no particles of undissolved iron oxide, while the SS sample possesses larger grain sizes. Undissolved iron oxides are observed in both 14.5 mol% samples, while the contrast between those particles and the surrounding YSZ matrix is much weaker for the CP samples due to the larger amount of iron dissolved in the YSZ.

For the CP material, incremental increases in iron content up to the solubility limit are wholly incorporated into the 8YSZ and create additional redox centers that are thermodynamically similar to those already present; further, these active species are accessible and able to participate in chemical reactions due to facile oxygen ion transport through the 8YSZ matrix. The behavior observed above the solubility limit can be rationalized with the reasonable assumption that the distinct iron oxide particles distributed through the YSZ matrix behave similarly to isolated iron oxide particles. First, the reaction thermodynamics of these particles is different from the dissolved iron. Second, and more importantly for these evaluations where the reaction is thermodynamically driven by a reactive sweep, significant kinetic limitations impact these particles. Oxygen transport though the iron oxides is limited under the prevailing conditions, and any redox reaction is confined to particle surface and near-surface (or YSZ/iron oxide interface) regions, where near-surface denotes regions within only a few microns of the interface. It has been shown that reduced iron oxide particles of <*ca.* 5 μm diameter can be re-oxidized during the 10 h of CO_2_ exposure, while larger particles are only partially converted.^39^ Further, larger particles that form at higher loadings are not only less reactive but subsume smaller particles on the time scale of an individual thermochemical cycle.^38^ This process decreases particle utilization because it increases the fraction of their volume that is too remote from the surface to participate in the reaction. In addition, these larger particles or collections of larger particles may hinder oxygen transport in 8YSZ through a physical blocking mechanism.

Iron incorporation into SS materials mirrors that of CP materials only to a loading of below 4.5 mol% Fe. Above that, a fraction of the iron remains in a less active form, presumably as the oxide, even though the iron loading is less than half of the solubility limit. Further, re-oxidation is insensitive to higher loadings, varying only from 0.14 to 0.17 mass% over the range 7.4–27.6 mol% Fe. These observations are consistent with a complex sample evolution that results in two somewhat distinct stages for incorporating Fe into the YSZ in SS materials. Upon initial heating, there is a large driving force for iron to incorporate into the YSZ, and the near-interface regions of the YSZ particles are populated with Fe. The extent of dissolution/distribution may be more limited than one would anticipate from the solubility data; Molin *et al.*^44^ suggest that Fe solubility in 8YSZ is diminished in grain boundary regions relative to the bulk, while according to de Ridder *et al.*^45^ a chemically unique diffusion barrier may form in the interfaces between iron oxide and YSZ. Evidently, under our conditions, this initial stage produces active Fe species that fully mirror that of the CP samples only up to a nominal (*i.e.* averaged over the bulk, not a local concentration) loading of about 4.5 mol%, above which an increasing fraction of the iron segregates to less active species, with the incorporation of Fe into active species appearing to saturate at about 7.4 mol% loading.

This apparent saturation is consistent with the sample transitioning into a second, mass transfer limited stage. Once the population of the surface or interface region occurs, subsequent incorporation of Fe is limited by the rate at which the active dissolved Fe diffuses into the interior regions of the YSZ particle and is replaced at the grain surface. The diffusion coefficient for Fe in 9.5YSZ has been reported as *ca.* 1 × 10^−19^ (4.4 × 10^−20^) m^2^ s^−1^ at 1400 (1300) °C.^46^ This corresponds to a diffusion length over 40 hours of only 0.25 μm at 1400 °C. While this is similar to average particle diameters for the commercial 8YSZ powder,^47,48^ the grains of YSZ in the SS materials after sintering are typically larger than 10 μm in diameter.^38^ In fact, hematite functions as a sinter aid for 8YSZ, beginning at temperatures < 1000 °C and approaching completion at 1350 °C.^49^ Thus, the YSZ particle growth is rapid relative to Fe diffusion. Simultaneously with the sintering of YSZ, the small and well-dispersed iron oxide particles initially present in the sample rapidly migrate and coalesce into larger particles; EDS indicates this process is facilitated by redox cycling with major changes observable after only a few cycles.^38^ Thus, the already slow diffusion of active Fe into YSZ is made more sluggish by physical changes in the samples that are virtually unavoidable during the preparation process.

It is important to remember that the thermodynamics governing the solid compositions and properties of this or any system are unmoved by the preparation details. Thus, it is reasonable to expect that CP and SS materials of similar composition will evolve over time towards a similar end state, particularly since the chemistry of interest requires high temperatures which can provide the energy to overcome kinetic barriers. Over the long term, the chemical homogeneity of the SS samples will improve, and the large iron oxide grains will gradually dissolve into the YSZ matrix up to the solubility limit. Similarly, the non-solubilized iron oxide particles in the CP samples will continue to agglomerate and grow, contributing ever less to the redox reaction.^38^ Note, however, that in this study the cycle-to-cycle behavior of samples subjected to multiple thermochemical redox cycles in the TGA varies only minimally, *i.e.*, changes of significance are occurring at a very slow rate. Indeed, taking grain growth into account, the diffusivity data quoted above would suggest that the Fe migration process would continue over a time scale of years to decades at 1400 °C. Thus, the differences between these samples can be expected to persist over practical time periods and treated as representing a pseudo-equilibrium capable of providing data representing steady- or near-steady-state conditions.

#### First cycle *versus* subsequent cycles

The first cycle of each TGA run resulted in net mass loss for the fresh material (*e.g.*, Fig. 1A), while each second and subsequent thermochemical cycle resulted in a net-zero mass change, provided CO_2_ was used consistently as the oxidant. Re-oxidation of a sample using air restores the sample to a state resembling the “freshly calcined” material. The net mass loss during the first TGA cycle increased with iron content; Fig. 1C.

To understand this behavior, consider first the low Fe-loading portion of the curves where all the Fe is dissolved into the YSZ matrix, *i.e.*, Fe contents < 4.5 and 9.4 mol% for SS and CP samples, respectively. The data in this region are consistent with an understanding that the incorporation of Fe into the YSZ creates a distinct single-phase material that is capable of adjusting its bulk oxidation state to achieve thermodynamic equilibrium with its environment. That is, within limits defined by stoichiometry or materials stability, the Fe^2+^ : Fe^3+^ ratio in the material changes as a function of the partial pressure of oxygen (*p*_O_2__) in the adjacent gas phase. The mass losses in this region for the two materials correspond to Fe^2+^ : Fe^3+^ ratios that are in equilibrium with a *p*_O_2__ of 4.6 × 10^−5^ atm, the *p*_O_2__ that results from CO_2_ thermolysis at 1100 °C. Note that the data in this region exhibit curvature. This means that the equilibrium Fe^2+^ : Fe^3+^ ratio, and hence the thermodynamics of the material, varies as a function of Fe loading. Note also that the data for the two sample types overlay one another at Fe loadings < 4.5 mol%. That is, the array of active Fe species produced by the two preparation techniques are thermodynamically indistinguishable from one another in the limit of low Fe content, consistent with the discussion of Fig. 1B. As a point of reference, in all cases, less than 80% of the Fe in solid solution participates in the standard redox cycle examined here, and utilization is approximately inversely proportional to iron loading.

Now consider the excess Fe (insolubilized Fe) regions of the curves. Guidelines provided in Fig. 1C illustrate the linear increase in first cycle mass loss with Fe content for the two types of material in this region. This behavior results from the basic thermodynamics of iron oxide. Prior to the first cycle, the materials were oxidized in air at 1450 °C where the thermodynamically stable state is Fe_2_O_3_, *i.e.*, all iron is fully oxidized to Fe^3+^. Thermodynamics further indicates that Fe_2_O_3_ will reduce stoichiometrically to FeO (Fe^2+^) under low oxygen partial pressures at 1400 °C. However, also according to thermodynamics, CO_2_ cannot oxidize FeO to Fe_2_O_3_, but rather should stoichiometrically yield the intermediate mixed valence phase, Fe_3_O_4_, as shown earlier.^17^ The dashed line in Fig. 1C indicates the predicted behavior if Fe in excess of 9.4 mol% conformed completely to thermodynamic expectations, transforming fully from Fe_2_O_3_ to FeO and back to Fe_3_O_4_ during the course of the first cycle, while the dissolved Fe cycled stoichiometrically from Fe^3+^ to Fe^2+^ and back to Fe^3+^. The guidelines in the figure closely parallel one another and the dashed line, thereby confirming that excess Fe in these samples conforms to expectations. It is also an indication that at this stage in their evolution, the iron oxide particles in the samples remain small enough to fully participate in the redox reaction over the time frames studied. The offset in the guidelines shows that, above 5 mol%, the SS materials have a larger fraction of undissolved iron than their CP counterparts.

#### Reaction rates and short-term aging

The rates of thermal reduction of these materials can be quantified using TGA, even though thermal reduction occurs concurrently with the temperature ramp up to the chosen reduction temperature (*i.e.*, non-isothermal conditions), as shown by Scheffe, *et al.*,^50^ and Allen, *et al.*^51^ In other words, the materials properties may be distinguished from instrument effects. Here we focus on the isothermal re-oxidation rates as an indicator of overall material performance, as these tend to be slower than reduction and, as such, may significantly impact scaled-up process metrics including throughput and material inventories.

In order to estimate the rates of re-oxidation of thermally-reduced ferrite materials, multi-cycle TGA experiments were carried out wherein the specimen was first thermally reduced under Ar at 1400 °C then cooled to the re-oxidation temperature and allowed to stabilize at that temperature for 15 min prior to introduction of CO_2_ for 30 min. This redox cycle was repeated sequentially (typically 5 times) to enable any short-term aging or history-dependent phenomena to be identified. In no case did the extent or rate of reaction vary significantly or systematically between cycles, notwithstanding the aforementioned increased mass loss during the first reduction cycle, indicating the absence of significant short-term aging phenomena. This procedure captures an initial rate; the solid sample does not approach the equilibrium re-oxidation extent during this 30 minute period.

**Fig. 2:**
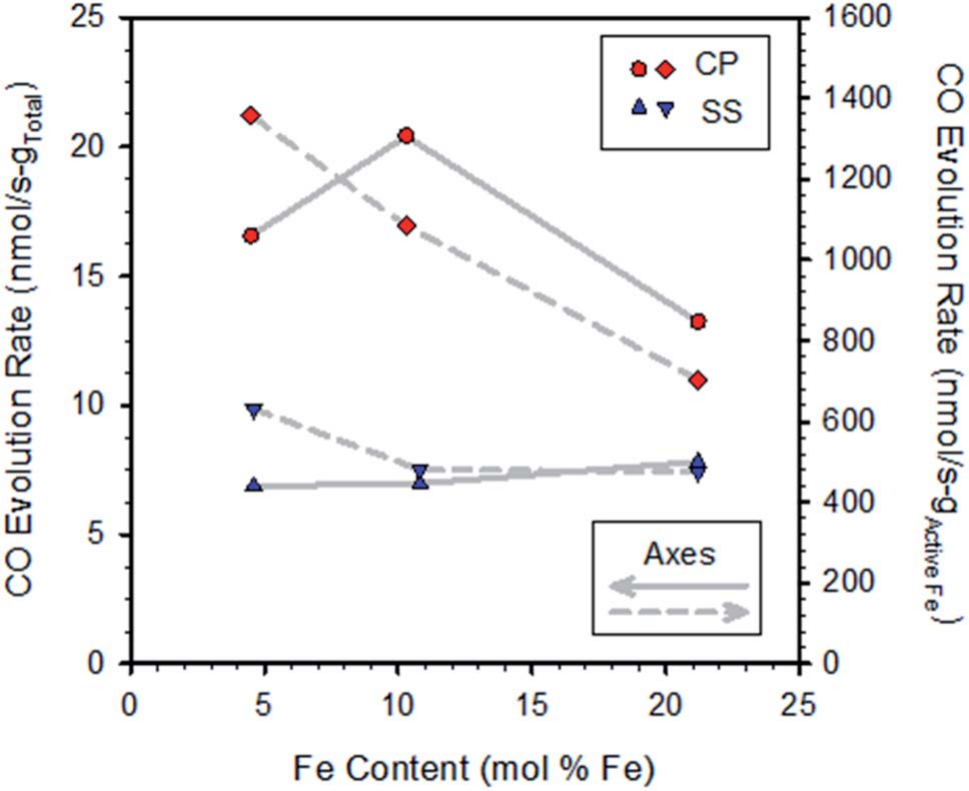
Rates of CO production for CP and SS samples (from Fig. S6†) as a function of iron content. Rates were calculated by linear regression over the first approx. 20 minutes of exposure to CO_2_ and were normalized to the total sample weight (left axis) and redox active Fe contents of the specimens (right axis).

Representative multi-cycle TGA plots are shown in the ESI, Section S4.† Fig. S6† compares data extracted from TGA multi-cycle runs recorded on pairs of samples synthesized with the same Fe content by either the SS or CP route. The materials prepared by CP react with CO_2_ more rapidly than those prepared by the SS method, and all SS-samples exhibit similar rates of mass increase. For the CP samples the sequence of re-oxidation rates *versus* content was: 10.3 > 4.5 > 21.2 mol% Fe. One might anticipate the rate of mass increase would be proportional to the Fe content, but clearly this is not the case (Fig. S6†). To explore this further, oxidation rates calculated from the curves in Fig. S6† are shown in Fig. 2. Normalizing the rates to total sample mass verifies there is no obvious trend with increasing Fe content (left axis).

Normalizing the rates to the redox active iron content (that is, the amount of iron found to cycle between reduced (Fe^2+^ or FeO) and oxidized (Fe^3+^ or Fe_3_O_4_) based on TGA data) reveals a pattern of decreasing rates with increasing Fe loading (Fig. 2, right axis; normalizing to total iron content gives a similar result, not shown). That is, the oxidation rates of the active iron species are largest at the lowest loadings and decrease as loadings increase. This result is particularly pronounced for the CP samples. From the previous discussions, we expect that all samples, with the exception of the CP sample with the lowest Fe content, contain some undissolved Fe and that the fraction of undissolved iron increases with loading. Thus, the results implicate the presence of bulk iron-oxide particles as being detrimental to the oxidation rate, presumably by increasing overall resistance to oxide ion diffusion through the material matrix, as well as slower kinetics of reaction of undissolved Fe compared to dissolved Fe.

### Effect of yttria content in YSZ on CDS activity of ferrite/YSZ materials

**Fig. 3:**
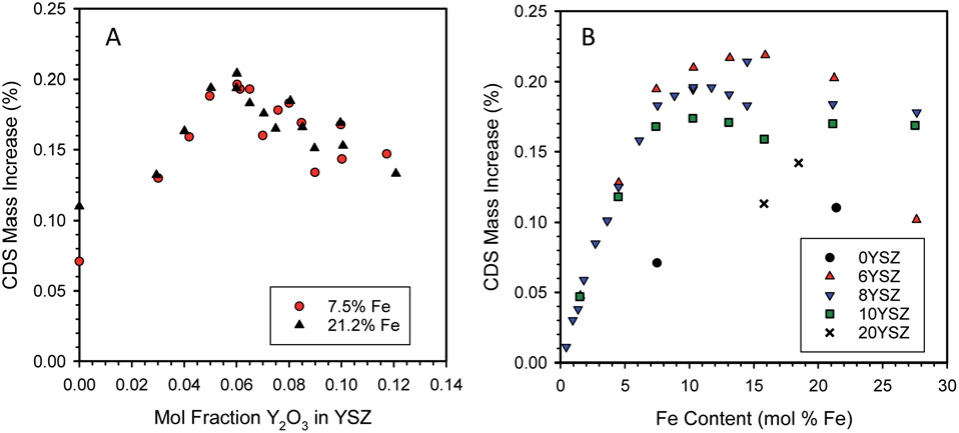
Effect of mole fraction of yttria in YSZ on the reactivity of ferrite/YSZ materials containing 7.5 or 21.2 mol% Fe (A). Effect of ferrite loading on the reactivity of ferrite/YSZ materials containing 0, 6, 8, 10, or 20 mol% yttria (B).

Samples containing a range of yttria and iron contents were prepared by the CP technique, pressed into discs, and heat-treated as described earlier. Fig. 3A shows the effect of varying the yttria content of Fe/YSZ materials on CDS properties for two distinct iron loadings (7.5 and 21.2 mol% Fe). A maximum in redox capacity of the materials is observed at an yttria content of approximately 6 mol% (expressed as mol% of the yttria and zirconia components only, *i.e.*, not including the iron contribution). However, there appears to be little difference in performance between the two Fe loadings at comparable yttria levels.

Characterization of the two sample sets used to generate the data in Fig. 3A by X-ray diffraction (XRD) at room temperature revealed a change in structure of the zirconia phase from monoclinic at low yttria content (below 6 mol% Y_2_O_3_) to cubic at higher yttria contents (6 mol% Y_2_O_3_ and above, see ESI, Section S5†). These observations agree with the literature for undoped YSZ^52^ and Fe-doped YSZ.^53^ For samples containing 21.2 mol% Fe, un-dissolved hematite (Fe_2_O_3_) was observed in all samples, as expected. At 7.5 mol% Fe only XRD peaks for zirconia were observed. Note that even though Fe must have been solubilized into the YSZ at low Y contents, at neither Fe loading did the presence of iron stabilize the cubic phase.

To further examine the interplay between yttria and Fe concentrations, additional samples were prepared with fixed Y and variable Fe contents. The resulting data set is presented in Fig. 3B. As before, the 6YSZ Fe-loaded materials exhibit the highest redox capacities, except at the very highest Fe loading. Up to Fe loadings of ∼ 5 mol%, all samples behave similarly, but above that loading the redox capacity follows the general order 6YSZ > 8YSZ > 10YSZ > 20YSZ > 0YSZ. Note that for 6, 8, and 10YSZ, redox capacities for the 7.5 mol% Fe are similar to those of 21.2 mol% Fe, suggesting the similarity between the two data sets in Fig. 3A is likely coincidental and of limited significance. Given that the data represent the equilibrium or near-equilibrium condition, the differences between the samples, particularly those of 6, 8, and 10YSZ, are unlikely the result of transport considerations. This is particularly true considering self-diffusion of oxygen in YSZ is maximum at an yttria content of approximately 10 mol%,^54^ so enhanced oxygen mobility in 6YSZ compared to 8YSZ or 10YSZ is unlikely. Based on SEM observations, Ratkje and Tomii reported the grain size in YSZ to increase from 1 μm for Y content of 5 mol% or lower, to 18 μm for 8 mol% Y,^55^ all under identical sintering conditions. Thus, changes in grain size of the iron-loaded YSZ with varying yttria content may be a factor in defining their thermochemical properties. The effect of dissolved Fe on the electronic structure, hence the oxygen mobility, in these systems is a factor that still must be investigated.

One possible explanation for the enhanced activity of 6YSZ relative to other compositions is the increased solubility of ferrite compared to the higher yttria-loaded materials. The point at which the CDS mass increase in Fig. 3B plateaus increases from 10.3 to 11.7 to 13.1 mol% Fe as the yttria content decreases from 10 to 8 to 6 mol% yttria, respectively. The XRD analysis of these samples (Fig. S7 and S8, insets, ESI†) shows a trend of increasing lattice parameter with increasing yttria loading, consistent with the literature;^56^ however a similar trend could be caused by decreasing levels of dissolved iron,^37^ thus the XRD results are inconclusive without more detailed investigation. If an inverse relationship between iron solubility and yttria content were the only factor at play, one would not expect the 6YSZ and 8YSZ data to begin to diverge below the solubility limit of iron in 8YSZ. Also, as described above, in all cases a large fraction of the dissolved Fe does not participate in the redox reaction at these conditions, so an increase in the solubilized amount is not necessarily required to realize an increase in the redox capacity. Along this line of reasoning, an alternative explanation is that the thermodynamics of the dissolved Fe vary in response to the yttria content. The data in this case would suggest that, when dissolved at higher loadings, Fe^2+^ present in 6YSZ is easier to re-oxidize (less thermodynamically stable) than Fe^2+^ in 10YSZ. These possibilities cannot be easily confirmed and differentiated between without detailed examination of the solid-state thermodynamics of this system, possibly including an examination of the Fe solubility as a function yttria content, oxidation state, and temperature.

### Effect of re-oxidation temperature

**Fig. 4:**
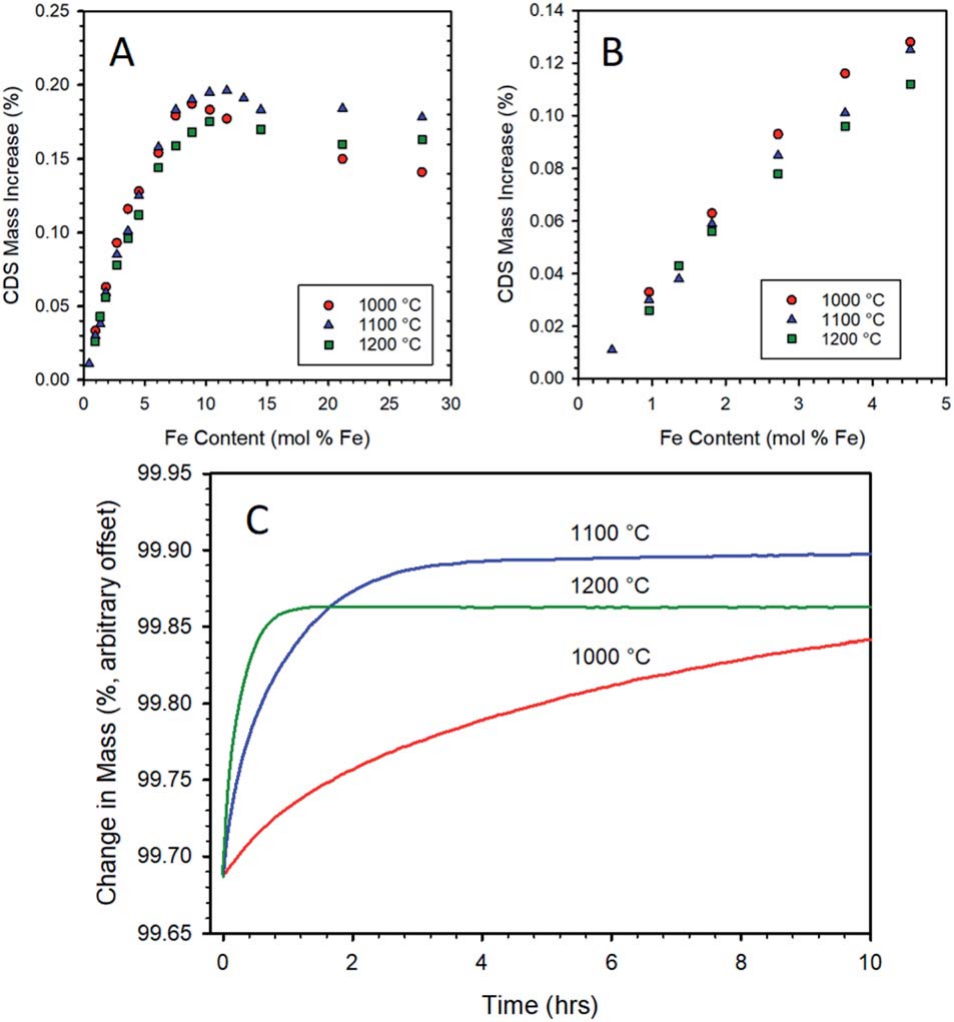
Mass increase during re-oxidation under CO_2_ as a function of iron loading in 8YSZ for CP samples as measured by TGA (A). Samples were thermally reduced at 1400 °C for 5 h, then re-oxidized at 1000 °C (15 h), 1100 °C (10 h), or 1200 °C (10 h). Expanded view of data for low iron-loaded samples (B). Comparison of TGA curves for a 10.3 mol% Fe/8YSZ sample prepared by CP after 1400 °C thermal reduction (5 h) followed by re-oxidation under CO_2_ at 1000 °C, 1100 °C, and 1200 °C (C). Only the isothermal re-oxidation step (first 10 h) is shown.

Samples prepared by CP with various Fe contents in 8YSZ were re-oxidized with CO_2_ at 1000, 1100, and 1200 °C after a standard thermal reduction step, *viz.* 5 h at 1400 °C under Ar. Data for the three re-oxidation temperatures show some common features, as seen in Fig. 4. The mass gain upon re-oxidation increases linearly, within experimental error, as the iron loading in the sample increases up to approximately 3 mol% (Fig. 4A and B). Thereafter, a slight decrease in slope occurs and the mass gain continues to increase in an almost linear fashion until an iron loading of approximately 9–11 mol%. At loadings above this level, the mass gain upon re-oxidation ceases to rise, and even begins to decrease with further increase in the iron loading. The data collected at 1000 and 1200 °C, follow the pattern previously established at 1100 °C, *vide supra*.

At iron loadings below 5 mol%, the maximum mass gain upon re-oxidation was exhibited by the data recorded at a re-oxidation temperature of 1000 °C (Fig. 4B). This is to be expected from a thermodynamic point of view, as lower temperatures favor the oxidation of iron. At low iron loadings, it appears that the dissolved iron can be re-oxidized at a sufficient rate at 1000 °C to give the expected increase in equilibrium re-oxidation extent compared to the higher re-oxidation temperatures. However, once the iron loading exceeded 5 mol% the overall rate of re-oxidation is suppressed to such an extent (see for example 10.3 mol% Fe in Fig. 4C) that even a 15 h exposure to CO_2_ at 1000 °C does not enable the material to reach a steady state extent of re-oxidation. Therefore, above this point the data for this material does not represent a thermodynamic equilibrium oxidation extent, but rather that achieved after 15 hours. This accounts for the apparent decrease in performance relative to the 1100 and 1200 °C conditions. Note that once the iron loading exceeds 9 mol% (*i.e.*, near the experimentally determined solubility limit for Fe in 8YSZ), there is a significant drop off due to incomplete re-oxidation. This is an indication that the oxidation of, or transport through, the un-dissolved bulk iron oxide particles is a more highly activated process than the oxidation of the solubilized species. The experiments in which re-oxidation was carried out at 1100 and 1200 °C for 10 h did result in the samples reaching a steady state (Fig. 4C). Comparing these two sets of data highlights the shift in equilibrium with temperature since the 1200 °C re-oxidation mass change data lies consistently below that recorded at 1100 °C. Considering all three data sets illustrates the trade-off between reaction thermodynamics (maximizing reaction extent of the solid and the CO content of the product gasses) and reaction kinetics which has important implications for process efficiency and viability.^57^

## Conclusions

An iron oxide/yttria-stabilized zirconia (YSZ) composite was utilized as a material to reduce (“re-energize”) carbon dioxide to carbon monoxide *via* thermochemical cycling. The thermochemical cycle involves reducing the iron species under inert atmosphere at around 1400 °C and re-oxidizing it with CO_2_ at temperatures between 1000 and 1200 °C. The performance of materials prepared by two methods, co-precipitation and solid-state synthesis, was compared. In both cases Fe dissolves into the YSZ matrix up to a limit determined by thermodynamic (solubility) or physical (slow reaction and diffusion into large grains) limits. Iron present in quantities beyond the solid solubility maximum segregates into iron oxide particulates. While thermodynamics will drive the materials prepared by these two routes towards a common end state, the rate of change is such that the differences exhibited by the materials appear to be “locked in” over a practical time frame.

The co-precipitated samples have a more uniform distribution of Fe throughout the material and in general exhibit greater redox capacity and faster rates of re-oxidation than solid state-synthesized materials of comparable composition. In all cases the extent of redox reaction varies in an approximately linear fashion with iron loading at low concentrations. Once the iron loading exceeds *ca*. 4 mol% for SS samples or 9 mol% for the CP samples, the extent of the redox reaction no longer scales linearly with loading. In the case of CP, the performance of the materials reaches a peak and even decreases at high iron content (>*ca*. 10 mol% Fe), while the SS samples continue to show small increases up to the highest Fe loadings. The general behavior can be ascribed to increasing amounts of undissolved iron oxide which (a) has different thermodynamic characteristics than dissolved iron, (b) is less reactive (subject to a strong transport limitation) than iron dissolved into the YSZ matrix, and (c) contributes to oxide ion diffusion resistance through the material as a whole. Differences between the samples are linked to differences in homogeneity and distribution of Fe between phases, and the relative impact of the above effects.

Zirconia adopts a monoclinic structure at room temperature, independent of Fe loading, for yttria contents below 6 mol%; at 6 mol% and above only the cubic structure is observed. There appears to be an optimum yttria content of *ca.* 6 mol% (in zirconia) at which the redox capacity of Fe in the composite system is maximized. Roughly a 10% and 30% increase in redox capacity was realized over 8YSZ and 10YSZ respectively for this composition. This is possibly due to a variation in Fe solubility with Y content, a variation in thermodynamics between the materials, or a combination of both.

As expected from equilibrium considerations, after a consistent reduction protocol, the extent of re-oxidation was greater at 1000 °C than higher temperatures provided the iron loading was below 5 mol%. For higher iron loadings, the rate of re-oxidation at 1000 °C was so sluggish that full re-oxidation was not achieved, even after 15 h exposure to CO_2_. Exposure to CO_2_ at 1200 °C resulted in the fastest rate of re-oxidation, but a lower extent of re-oxidation than could be achieved at either 1100 °C or 1000 °C for all but the very highest Fe loadings.

## Conflicts of interest

There are no conflicts to declare.

## Supplementary Material

RA-011-D0RA08589H-s001
