# Supplementary material for: Compositional and operational impacts on the thermochemical reduction of CO2 to CO by iron oxide/yttria-stabilized zirconia
Source: RSC Adv. 2021 Jan 5;11(3):1493–502. doi: 10.1039/d0ra08589h (PMC8693632; doi:10.1039/d0ra08589h)
Supplement: RA-011-D0RA08589H-s001 [file RA-011-D0RA08589H-s001.pdf]

Electronic Supplementary Material (ESI) for RSC Advances.
This journal is © The Royal Society of Chemistry 2020

Electronic Supplementary Information

Compositional and operational impacts on the thermochemical reduction of CO_2 to CO by iron oxide/yttria-stabilized zirconia

Eric N. Coker,* Andrea Ambrosini, and James E. Miller†

Sandia National Laboratories, PO Box 5800, Albuquerque, NM 87185-1411, USA.

†Current Address: LightWorks®, Arizona State University, Tempe, AZ 85281, USA.

*encoker@sandia.gov

S1, S2) Control experiments – effects of sample porosity and mass transfer limitation

Electron microscopy and porosity measurements have indicated that CP pellets typically possess greater porosity than their SS counterparts[1] when synthesized as described in the paper. Thus, to ensure that the reported conversions are not influenced by particle size and/or mass transfer limitations, two different verification procedures were undertaken. First, an alternate strategy for forming discs of CP materials with porosity comparable with that of the SS discs, was investigated (see Section S1 for details). TGA experiments showed similar steady-state (pseudo-equilibrium) extents of reaction and rates of re-oxidation independent of the disc-forming protocol used for the CP samples, suggesting that porosity is not a major factor contributing to differences in re-oxidation properties. Second, several identical TGA cycles were performed on CP specimens of successively greater mass of the same material. In each case the specimen was in the form of a single fragment of a pressed disc. Only in the case of the highest iron loading (28 mol-%) was a slight decrease in the mass-% change upon re-oxidation observed when the sample mass exceeded 500 mg, see Section S2. For smaller sample masses, and for samples with lower iron loadings, there were no noticeable effects of sample mass on the measured conversion. All of the steady state data reported herein correspond to parameters wherein no size effects were observed.

S1) Alternative CP sintering strategy

A subset of CP powders was subjected to a low-temperature calcination step (600 °C in air) prior to adding binder and forming into discs. This pretreatment condenses hydroxyl groups in the as-precipitated material, forming oxide phases. The pretreatment decreases the mass loss that occurs during subsequent binder calcination and accordingly decreases the void volume (porosity) of the resulting disc. Comparison of the CDS mass gain for sample pairs prepared with and without this pretreatment (i.e. as described in the paper) reveals only minor and non-systematic differences in performance (Figure S1), verifying that the greater porosity of CP specimens cannot account for the improved conversion relative to SS specimens.

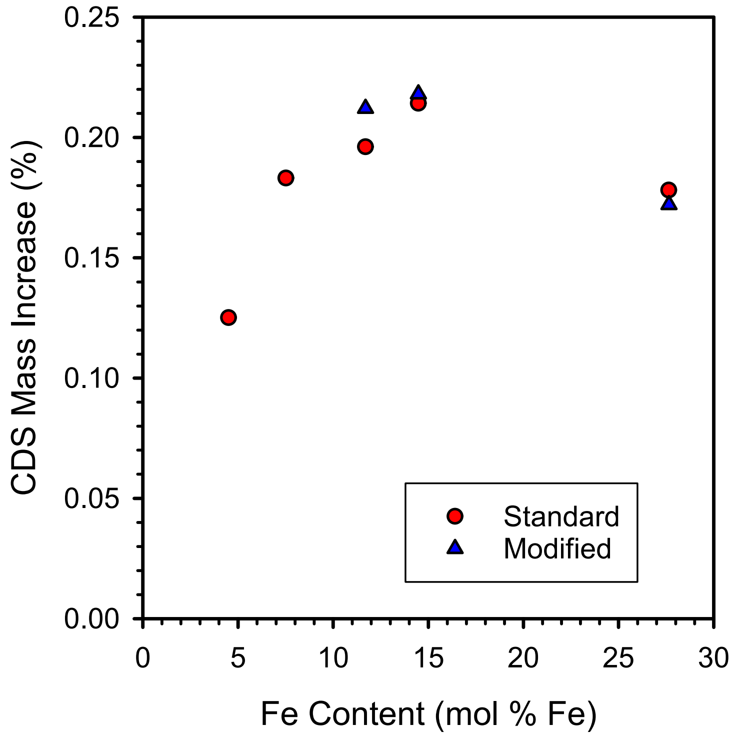

Figure S1. Comparison of CDS performance versus iron loading for pairs of samples prepared by standard or modified (see above) procedures.

S2) Mass transfer limitation study.

Specimens of differing mass were taken from CP samples of three distinct iron loadings (7.5, 14.5, and 27.7 mol-% Fe in 8YSZ), and were subjected to an identical TGA cycle including 5 hours thermal reduction (TR) at 1400 °C and 10 hours re-oxidation with CO_2 (CDS) at 1100 °C. The mass gain during CDS is plotted against the specimen mass in Figure S2. All data points lie close to the linear regression drawn through the data for the 14.5 mol-% Fe sample except for one, which is for the highest mass specimen of the highest Fe-loaded sample. This data point showed lower CDS mass increase than the linear regression, showing that only under this specific loading of active material is mass transfer limiting.

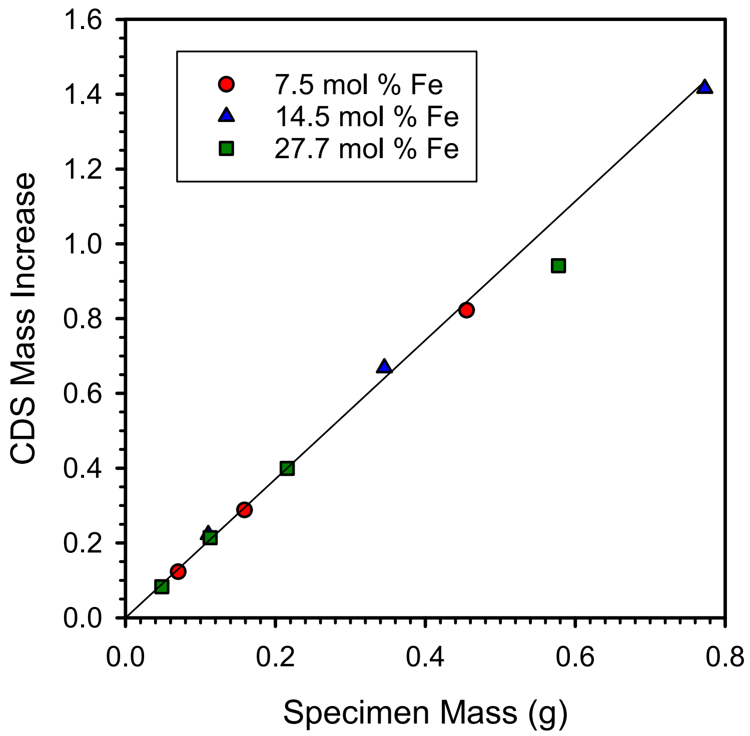

Figure S2. Absolute CDS mass increase as a function of specimen mass and iron oxide loading.

S3 SEM/EDS images of dense monolithic samples

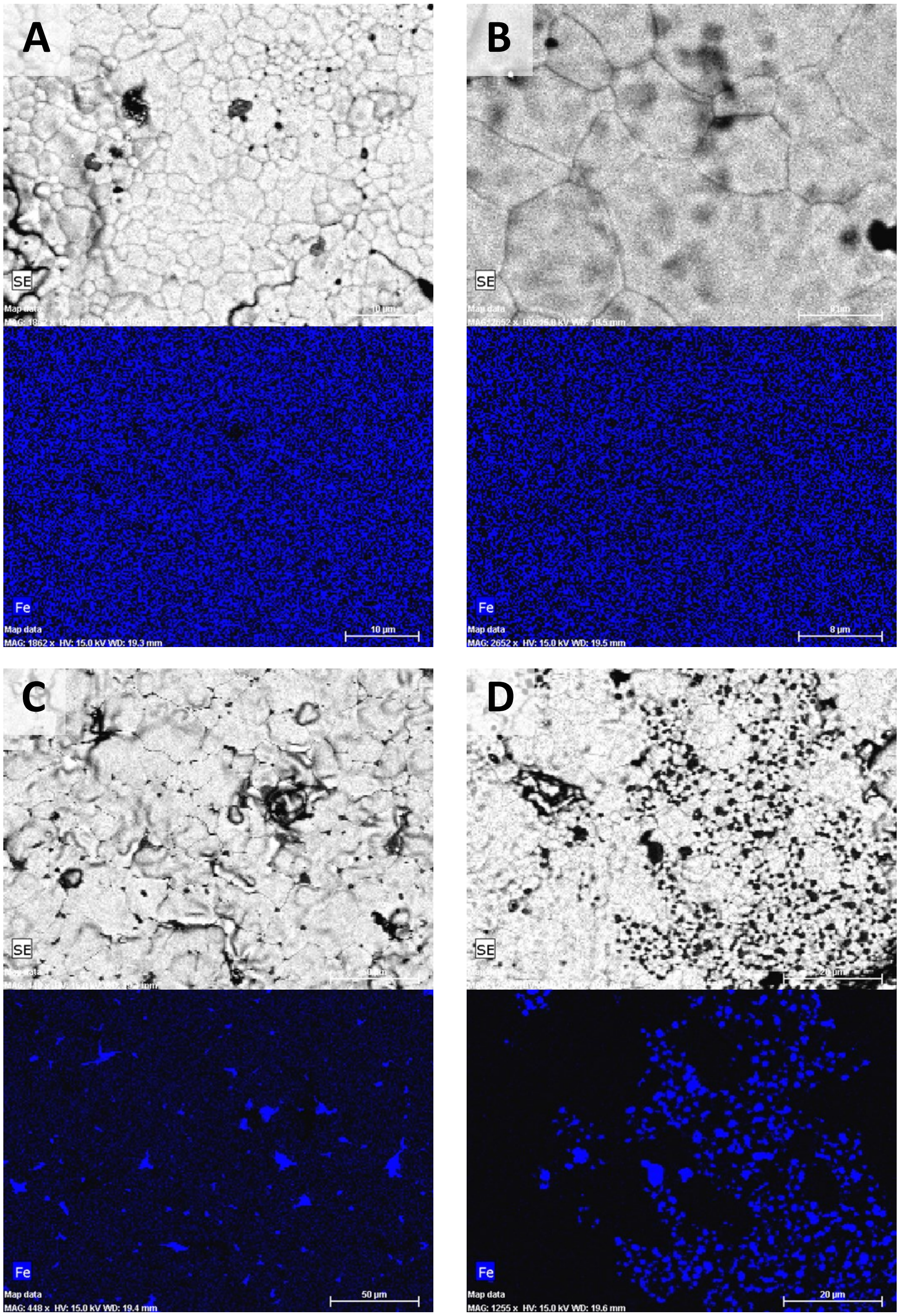

Figure S3. SEM secondary electron images and EDS maps for Fe in dense monolithic samples containing 4.5 mol-% iron (A, B), and 14.5 mol-% iron (C, D). Samples prepared via CP route (A, C) and SS route (B, D).

S4) Stability over multiple TGA cycles

The results of multi-cycle TGA on a representative pair of CP and SS specimens is shown in Figure S4. Figure S5 shows an overlay of the three 30-minute CO_2 re-oxidation steps, showing negligible difference in rate or extent of reaction between the three steps, verifying that the short-term ageing effects in these specimens are minimal. Figure S6 illustrates the differences in re-oxidation rates for three CP samples and three SS samples, taken from the second re-oxidation cycle in multi-cycle experiments.

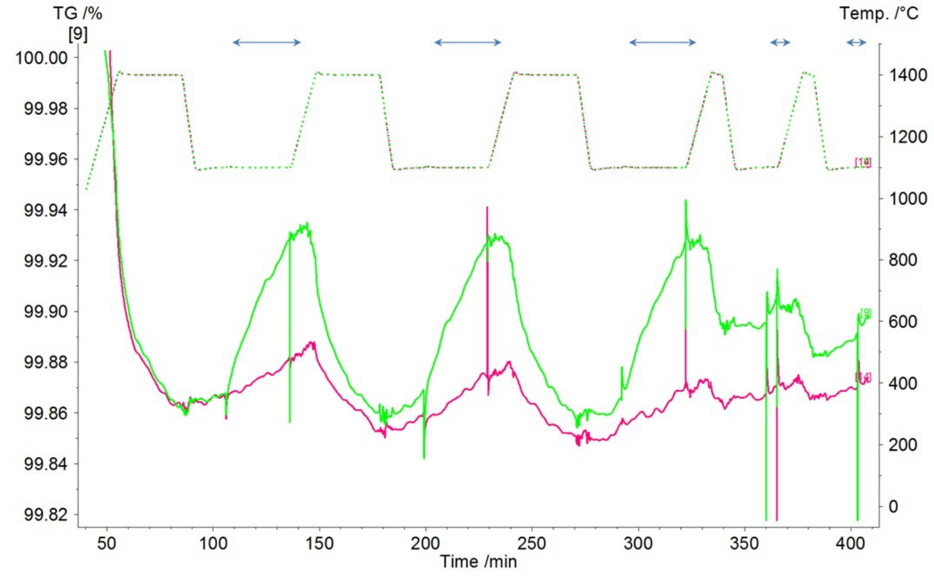

Figure S4. Multi-cycle TGA data for 10.5 mol-% Fe in 8YSZ prepared by CP (green) and SS (red) methods. Dotted curves show sample temperature. Double-headed horizontal arrows represent re-oxidation steps under CO_2, remainder of experiment is under argon.

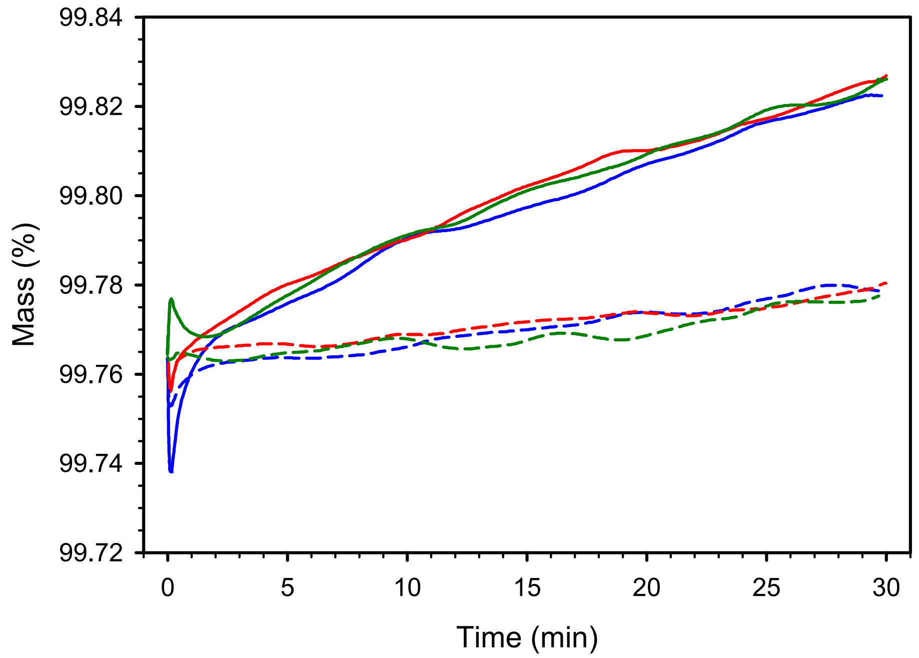

Figure S5. Overlay of re-oxidation performance for the three successive 30-minute isothermal CDS stages indicated in Figure S4. CP specimen (solid lines) and SS specimen (dashed lines).

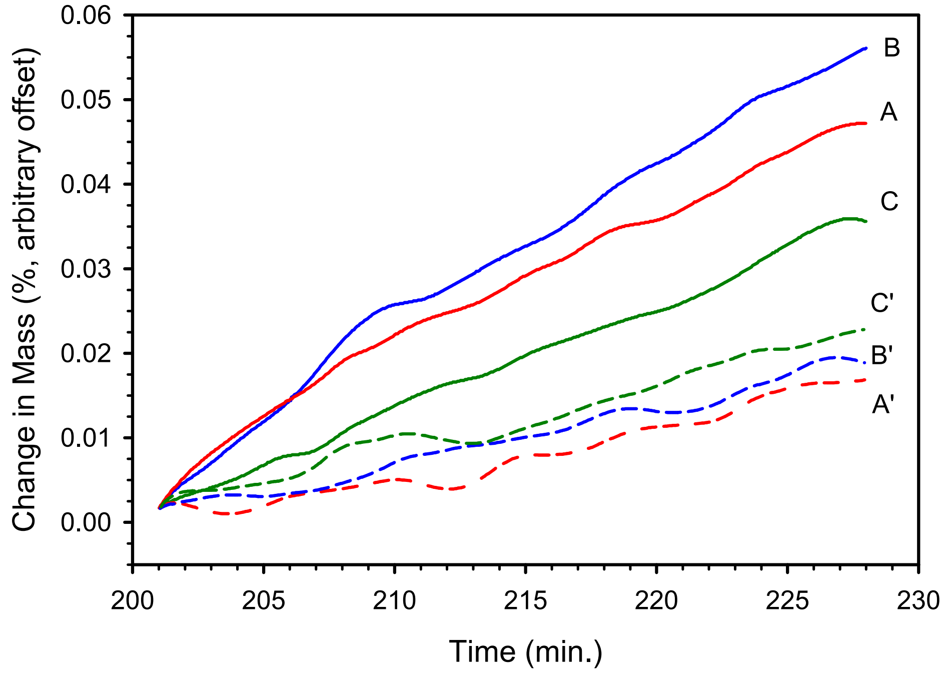

Figure S6. Mass gain during 2nd re-oxidation cycle of iron oxide/8YSZ containing 4.5 mol-% Fe (A, A′; red), 10.3 mol-% Fe (B, B′; blue), and 21.2 mol-% Fe (C, C′; green). Samples prepared via CP (A, B, C; solid lines) and SS (A′, B′, C′; dashed lines) methods.

S5) XRD of variable Y content YSZ samples

Room temperature XRD patterns of various ferrite/YSZ samples prepared with yttria contents between 3 and 12 mol-% prepared by the CP method are summarized in Figures S7 (7.5 mol-% Fe) and S8 (21.2 mol-% Fe). At yttria contents below 6 mol-% monoclinic zirconia, or a mixture of monoclinic and cubic (or tetragonal) zirconia are observed. At 6 mol-% Y and higher, only the cubic (or tetragonal) phase is observed, regardless of the iron concentration. The inset figures show a detail of the YSZ (111) peak for each yttria content. The trends seen here are consistent with other observations in the literature.[2]

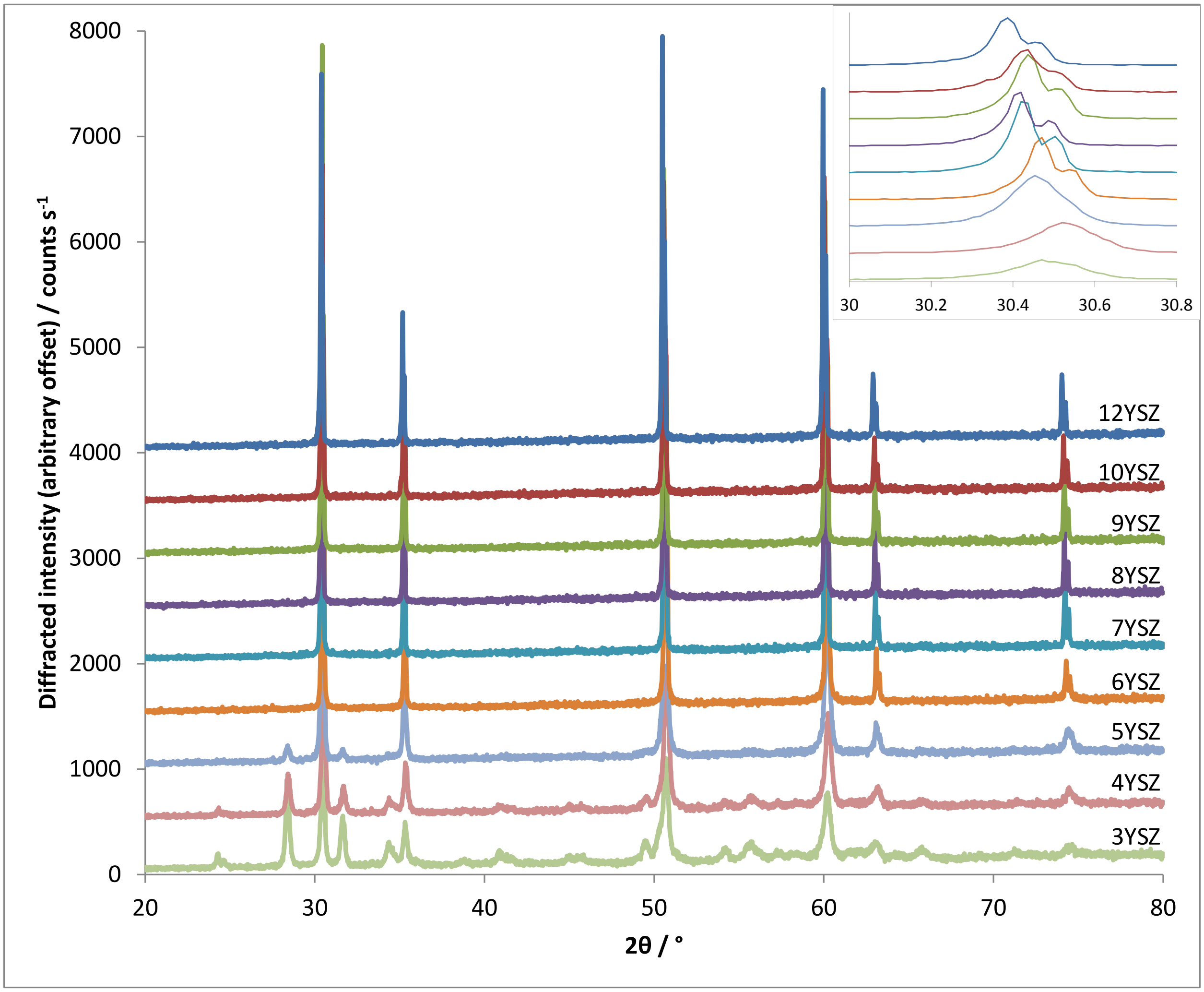

Figure S7. X-ray diffraction patterns for as-prepared specimens containing 7.5 mol-% Fe, and various yttria contents (indicated by labels on right). Only peaks arising from zirconia or yttria-stabilized zirconia are seen.

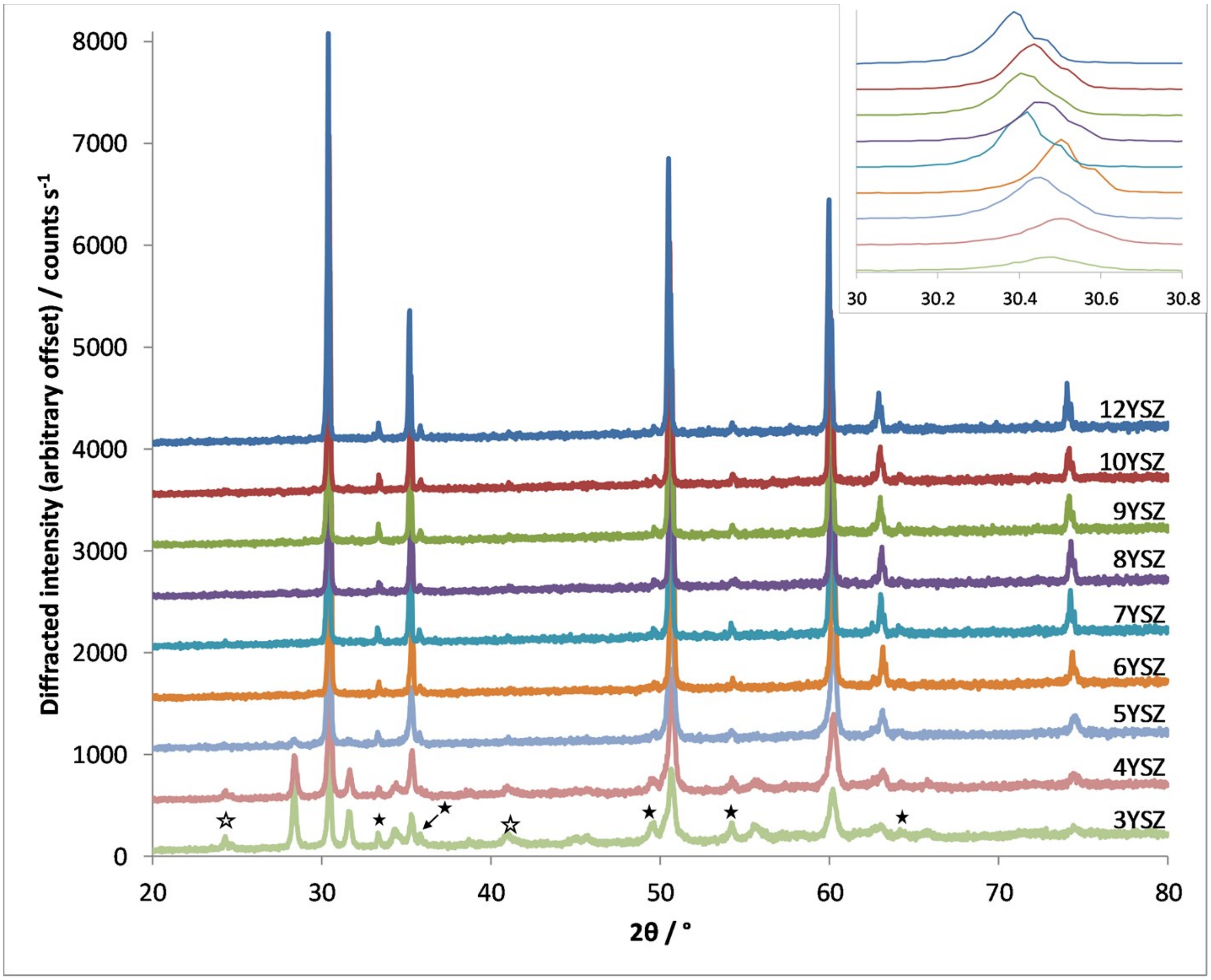

Figure S8. X-ray diffraction patterns for as-prepared specimens containing 21.2 mol-% Fe, and various yttria contents (indicated by labels on right). Filled stars designate peaks arising mainly from un-dissolved hematite (Fe_2O_3), while the peaks with open stars have significant overlapping signals from hematite and monoclinic zirconia. Unmarked peaks are attributable to zirconia (monoclinic, or yttria-stabilized cubic/tetragonal form).

References

1 E.N. Coker, J.A. Ohlhausen, A. Ambrosini and J.E. Miller, *J. Mater. Chem.*, 2012, **22(14)**, 6726-32.

2 S. Vasanthavel and S. Kannan, "Structural investigations on the tetragonal to cubic phase transformations in zirconia induced by progressive yttrium additions" *J. Phys. Chem. Solids* **112** (2018) 100–105.
